# Evaluation of REACH-TX: A Community-Based Approach to the REACH II Intervention

**DOI:** 10.1093/geroni/igz022

**Published:** 2019-08-23

**Authors:** Jinmyoung Cho, Susanna Luk-Jones, Donald R Smith, Alan B Stevens

**Affiliations:** 1 Center for Applied Health Research, Baylor Scott & White Health, Temple, Texas; 2 Center for Population Health & Aging, Texas A&M University Health Science Center, College Station; 3 Department of Environmental and Occupational Health, Texas A&M University Health Science Center, College Station; 4 Family Care Services Unit, Alzheimer’s Association North Central Texas Chapter, Fort Worth; 5 Area Agency on Aging, United Way of Tarrant County, Fort Worth, Texas; 6 College of Medicine, Texas A&M University Health Science Center, College Station

**Keywords:** REACH-TX, Caregivers for Alzheimer’s disease and dementia, Community-based support, Translational research

## Abstract

**Background and Objectives:**

Family caregiving interventions have been proven efficacious at reducing dementia caregiver’s stress and burden, yet translation of evidence-based interventions into community-based support service programs requires modification to the original intervention protocol. In collaboration with community partners, the REACH-TX program was developed based on the REACH II (Resources for Enhancing Alzheimer’s Caregiver Health) intervention. REACH-TX maintains the integrity of the multicomponent skill-based REACH II intervention but requires significantly fewer therapeutic contacts between the family caregiver and the dementia care specialist. This study presents an evaluation of REACH-TX implemented by the Alzheimer’s Association North Central Texas Chapter.

**Research Design and Methods:**

REACH-TX was provided to 1,522 caregivers between November 2011 and December 2017. The number of therapeutic contacts scheduled for caregivers was determined by the Risk Appraisal Measure (RAM) and ranged from 1 to 23. The rate of follow-up data on outcome measures collected was 59.0% (*n* = 898). All five domains of the REACH II quality-of-life measure (burden, depression, social support, self-care, and problem behaviors) were assessed at baseline and at 6 months. Caregivers (*n = 5*3) participating in the program more than once allowed us to investigate the long-term impact of the first exposure to REACH-TX and the value of repeating the program. Generalized linear models were used to assess changes in quality of life after adjusting for covariates.

**Results:**

Caregivers who completed the program showed significant improvements from baseline to 6 months on all five domains of quality of life, as evidenced by the follow-up data. Furthermore, caregivers who enrolled a second time in REACH-TX showed significant improvement in burden and social support scores.

**Discussion and Implications:**

This evaluation of REACH-TX suggests that REACH II evidence-based intervention can be translated into a valuable and sustainable community-based service for family caregivers. Additional translational research is needed to overcome the challenges of conducting standardized outcome assessments of caregiving services.

**Translational Significance**

REACH II, a six-month intervention shown to reduce caregiver burden in randomized control clinical trials, was modified in a community setting (named REACH-TX) to include the reduced in-person and telephone contacts between the caregiver and the Dementia Care Specialist. The REACH-TX was found to be effective in improving the quality of life including reducing caregiver burden when administered by a community agency.

As baby boomers enter their late 60s and beyond, the risk for Alzheimer’s disease and related dementia (ADRD) is elevated (Alzheimer’s Association, 2019). The number of people aged 65 and older with ADRD is expected to increase from 5.5 to 13.8 million by 2050 (Alzheimer’s Association, 2019). This increase will not only affect those persons directly, but also the millions of family members. In 2018, more than 16 million U.S. citizens provided a total of 18.5 billion hours of care for family members or close friends with ADRD (Alzheimer’s Association, 2019). Family caregivers for people with ADRD are more likely than other caregivers of older adults to assist with a variety of tasks, including activities of daily living (e.g., dressing, bathing, managing incontinence), coordination of care, and management of medical needs and symptoms (Czaja et al., 2018).

For several decades, a number of studies have found that the stress and burden associated with caregiving are common and negatively affect the health and well-being of family caregivers (Ory, Yee, Tennstedt, & Schulz, 2000; Schulz & Williamson, 1994; Vitaliano, Young, & Zhang, 2004). For example, studies, including a meta-analysis, have found caregivers have increased stress and depression as well as lower levels of well-being than noncaregivers (Pinquart & Sorensen, 2003). A recent report also indicated that most caregivers are unprepared for the caregiving role and experience adverse physical, social, and emotional consequences (National Academies of Sciences, Engineering, and Medicine [NASEM], 2016).

Numerous efforts to mitigate the negative consequences of the caregiving role and improve the quality of life (QOL) of caregivers have been developed. An estimated 200 interventions for family caregivers have been tested using randomized study designs, and most report positive effects with very few showing either no benefits or adverse effects (Gitlin, Marx, Stanley, & Hodgson, 2015). For example, the REACH II program, a multicomponent intervention, provided a range of strategies (e.g., education, skills training, social support, environmental modification) that has been extensively tested using randomized study designs (Belle et al., 2006; NASEM, 2016). Caregivers in the REACH II program showed significant improvement in burden, depression, health and self-care, social support, and management of patient behaviors (Belle et al., 2006). Specific features of REACH II are given in Table 1. Other family caregiving interventions have demonstrated benefits including reductions in nursing home placement (New York University Caregiver Intervention: Mittelman, Haley, Clay, & Roth, 2006), enhanced skills to manage functional dependence (Skills2Care: Gitlin et al., 2003), and implementation of effective care management (Partners in Dementia Care: Bass et al., 2014).

**Table 1.** Comparisons of REACH II and REACH-TX

Features	REACH II	REACH-TX
Design	Randomized control trial	Pre- and post-test
Activities	Assessment and feedback, education, skills training, referrals, support, problem-solving	
Content	• Safety	• Safety
	• Self-care	• Social support
	• Social support	• Stress management
	• Emotional well-being	• Pleasant events
	• Problem behaviors	• Healthy living
		• Understanding feelings
		• Skillful communication
		• Memory problems and behaviors
		• Legal and medical information
Delivery format	Nine in-home sessions and three telephone sessions with five structured tele-support group sessions	Two to six in-home therapeutic sessions with at least two phone calls based on Risk Appraisal Measure (RAM) score
Risk assessment	REACH II Risk Appraisal Measure using 139 total assessment items	Risk Appraisal Measure (RAM) using a 16-item measure (Czaja et al., 2009)
Materials and resources	Caregiver Resource Book: contains educational information about dementia and other relevant areas of caregiving	Caregiver’s Notebook: based on the original REACH II intervention manuals and reformatted to a resource that was familiar to consumers of support services
	Caregiver Network Computer Telephone Integration System (CTIS): a telephone-based system that enables caregivers to access basic information, referral and tips on different aspects of caregiving Behavioral prescriptions: specific behavioral prescriptions on targeted care recipient behaviors and/or issues related to communication and social support, including one- to two-page strategies that are action oriented and individualized to address a particular problem area	Family profile: included with the Caregiver’s Notebook to tailor the specific risks/needs of the caregiving family Supplementary handouts: a dementia care specialist provides a library of handouts and other resources that can supplement the content and further tailor the materials Support group referral: provided a list of support groups in their area and encouraged to participate as part of the social support intervention

Despite the broad base of evidence supporting the efficacy of caregiver interventions, few have been implemented in practice. Gitlin and colleagues (2015) identified that only 16 published works of translational studies have shown effectiveness in different settings, such as communities (REACH NC: Altpeter, Gwyther, Kennedy, Patterson, & Derence, 2015; REACH II: Lykens, Moayad, Biswas, Reyes-Ortiz, & Singh, 2014), the Area Agency on Aging (REACH OUT: Burgio et al., 2009), and health care settings, including hospitals (Family Caregiver Program: Stevens, Smith, Trickett, & McGhee, 2012). Common modifications include reductions in dosage (i.e., number of therapeutic sessions), changes in delivery mode, elimination of treatment components (e.g., technology application), and changes in interventionist training (Gitlin et al., 2015). Such modifications were intended to streamline complex interventions to fit the delivery environment. REACH OUT, for instance, is a translation of REACH II adapted to the Area Agencies on Aging (Burgio et al., 2009). The number and duration of therapeutic sessions were condensed into four home visits and three phone calls during a 4-month period. Significant improvements were found in caregiver burden, social support, frustration of caregiving, depression, caregiver health, and care recipient behavior problems (Burgio et al., 2009). However, to the best of our knowledge, there has been no further implementation of the REACH OUT intervention in other areas or settings, and there is no other available information on the sustainability of program itself.

## Purpose of the Study

Given the variety of evidence-based interventions that could potentially help dementia caregivers, it is necessary to ensure that these interventions maintain their effectiveness when translating them into a wide range of delivery settings. Translational studies, however, are still limited in part due to the extremely small portion of dementia caregivers in the U. S. who have access to proven interventions (Gitlin et al., 2015). Thus, a critical need exists for the translation, implementation, and evaluation of caregiver interventions across care and service-delivery settings. Such efforts are needed if the U. S. is to achieve “development and testing of dissemination and implementation strategies to enable reach and scaling up of proven programs and integration in existing systems of care” (NASEM, 2016, p. 180). In this context, the purpose of this study was to evaluate the impact of REACH-TX, a translation of the REACH II intervention for community-based organizations, and to show its sustainability. REACH-TX was implemented by the North Central Texas Alzheimer’s Association with funding from the United Way of Tarrant County, Texas. Evaluation activities reported in this study were conducted by Baylor Scott and White Health (BSWH).

## Method

### REACH-TX

REACH-TX is a community-based translation of REACH II, which was created by one of the coauthors and colleagues and tested in a health care setting (Belle et al., 2006; Stevens et al., 2012). Similar to REACH II, REACH-TX was designed to facilitate delivery of evidence-based skills training and support for dementia caregivers within community settings. REACH-TX is driven by the Stress Health Process Model (Schulz, 2000), which emphasizes interactions among the caregiver, his or her care recipient, and the physical and social environments by linking objective environmental stressors to health outcomes (Ory et al., 2000). REACH-TX provides assessment and feedback, education, skills training, referrals, problem-solving strategies, and support designed to help dementia caregivers reduce their stress and depression as well as improve their own capacity for self-care. As given in Table 1, although original REACH II focuses on five intervention components (safety, self-care, social support, emotional well-being, and problem behaviors), REACH-TX was reorganized into nine target components: safety, social support, stress management, pleasant events, healthy living, understanding feelings, skillful communication, memory problems and behaviors, and legal and medical information. This modification did not change the content of the intervention target components, but resulted in a more user-friendly format when compared to the original REACH II intervention materials. For REACH-TX, certified dementia care specialists (DCS; the community term for dementia interventionists), who were trained by the one of the coauthors, delivered the intervention for 6 months in English or Spanish via in-home and telephone sessions. The number of therapeutic home visits and key domain areas of focus for the caregiver were determined by the caregiver’s risk level using the Risk Appraisal Measure (RAM; Czaja et al., 2009). The RAM assessed personal/environmental challenges and needs that could contribute to negative outcomes for caregivers and increase the risk of placing a patient with dementia in an institutional setting (Czaja et al., 2009). The minimum number of therapeutic contacts for low-, medium-, and high-risk levels were 2, 4, and 6 contacts, respectively. The determination for cutoff values were driven by a number of considerations. First, there was existing evidence that other versions of the REACH protocol using two fewer sessions still delivered similar outcomes in health care settings (Stevens et al., 2012; Nichols et al., 2016). Second, maximizing the feasibility of program implementation through reduction of therapeutic contacts was a recurrent theme during planning discussions with community-based organizations charged with delivering the program. Finally, there were practical considerations for reducing the number of therapeutic contacts related to the funding constraints imposed by the funding agency and the need to service a sufficient number of clients to meet the initiatives broad goals. The number of therapeutic contacts was determined by the level of caregiver risk as indicated by the RAM.

Educational information and skill-training tools in REACH-TX were provided via *A Caregiver’s Notebook* (Stevens, Trickett, Smith, & Lancer, 2009), which contains problem-solving strategies and referral information (e.g., local caregiver support groups) on each component. DCSs developed a personalized “Family Profile” using the RAM to prioritize and address issues in domains showing the highest risk. Caregivers were also encouraged to attend support groups and were provided relevant information from library handouts and other resources. In addition, DCSs provided encouragement, validation, and empowerment regarding the target component topics. Supplementary Table 1 shows specifics of the intervention topics.

### Participants

Eligibility criteria for caregiver participants included residence in Tarrant County, Texas, and provision of at least 8 hr of care a week for a loved one with dementia living at home. The care recipient must have (i) required assistance with two or more activities of daily living or at least three instrumental activities of living and (ii) had a dementia diagnosis or experienced impairments in three of 10 memory functions as guided by the Alzheimer’s Association (2009). Recruitment occurred through referrals from local primary care clinics, home health providers, faith-based organizations, and community agencies. Staff of the Alzheimer’s Association North Central Chapter (AANCC) expanded new local collaborations and deepened existing collaborations to identify participants. REACH-TX was provided to 1,592 caregivers between November 2011 and December 2017.

### Procedures

After receiving referrals to the program from numerous community sources, AANCC staff implementing the REACH-TX program contacted potential participants to describe the program and conduct eligibility screening which included administration of the RAM. When it was determined that the participant was eligible and agreed to participate, basic demographic information was obtained over the phone and an initial home visit was scheduled within 2 weeks of the call. During the initial home visit, the program was reviewed and the initial assessment of QOL was administered before introducing the therapeutic content. Follow-up assessment of QOL was collected over the phone 6 months after program completion by a staff member other than the participant’s DCS.

### Measures

#### Personal characteristics

Caregiver demographic variables included age, sex, and race/ethnicity. Age was calculated based on each caregiver’s birth year and used as a continuous variable. For sex, a female was scored as 1. Race includes four categories: white/Caucasian, black/African American, Asian, American Indian/Alaska Native, Native Hawaiian/Other Pacific Islander, and multirace. Ethnicity was asked whether a participant is Hispanic/Latino or non-Hispanic/Latino. For statistical analysis, two dummy variables for race were created to compare non-Hispanic white or Caucasian and others and to compare Hispanic and others.

#### Risk appraisal measure

The RAM is a 16-item validated measure based on the REACH II 59-item baseline battery (Czaja et al., 2009). It targets six risk domains: depressive symptomatology, caregiver burden, self-care and healthy behaviors, social support, safety, and patient problem behaviors. Total possible scores range from 0 to 40 with higher scores indicating a higher-risk caregiver. The RAM has demonstrated adequate internal consistency for the entire scale (Cronbach’s α = .64) and convergent validity with other widely used measures, including the Center for Epidemiologic Studies Depression Scale (CES-D) and the Revised Memory and Problem Behavior Checklist. The RAM has also demonstrated similar measurement properties across ethnic and racial groups and has been used successfully in previous translations of REACH II (i.e., REACH VA and REACH OUT; Czaja et al., 2009). Internal consistency of the 16 items used in this study was .67.

#### Therapeutic contacts

Upon the confirmation of eligibility of caregivers for REACH-TX, a DCS administered the RAM to identify a caregiver’s overall risk level: a score of 0–12 denoted low risk; 13–19, moderate risk; and 20–40, high risk. Depending on the risk level, the DCS identified the targeted number of therapeutic home visits and key domain areas of focus for the caregiver. The DCS tracked the number of therapeutic sessions (i.e., home visits and telephone contacts) while the program was implemented. The number of therapeutic contacts ranges from 1 to 23.

#### REACH II QOL measures

Five caregiver QOL domains were measured: depression, caregiver burden, social support, self-care, and care recipient’s problem behavior (Belle et al., 2006).

##### Depression. 

The 10-item CES-D (Radloff, 1977) is scored on a scale of 0 (rarely or none of the time) to 3 (most or all of the time). Cronbach’s alpha of the CES-D is .88 for this study. The total score ranged from 0 to 30 with higher scores indicated greater depressive symptoms.

##### Caregiver burden. 

The Zarit Caregiver Burden Interview (ZCBI; Zarit, Reever, & Bach-Peterson, 1980) was used to assess caregiver burden. The ZCBI includes 12 items scored on a scale of 0 (never) to 4 (nearly always). The total scores of the ZCBI ranged from 0 to 48, and the internal consistency estimate of the ZCBI was .85. Higher scores indicated greater burden.

##### Social support. 

Social support was assessed with the eight-item Social Support Composite (Barrera, Sandler, & Ramsey, 1981; Krause, 1995). Responses were scored on a 4-point scale (0 = never to 3 = very often/always). Cronbach’s alpha of the social support is .85 for this study. A total score ranged from 0 to 24; higher scores indicated an increased level of social support.

##### Self-care. 

The 11-item measure on self-care scored questions on a yes (= 1) or no (= 0) scale to assess a caregiver’s diligence in looking after his or her own health (Belle et al., 2006). One question was sex specific (males asked about prostate examination and females asked about mammogram or Pap smear). Internal consistencies of the self-care measure are .49 for females and .57 for males. The total score ranged from 0 to 11 with higher scores indicating greater attention being given to one’s health and well-being.

##### Problem behavior. 

Three questions from the Revised Memory and Behavior Problems Checklist (Teri et al., 1992) assessed changes in problem behaviors of care recipients. Responses were scored on a yes (= 1) or no (= 0) scale. Cronbach’s alpha for this measure is .78. The total score ranged from 0 to 3; higher scores indicated substantial improvement in the loved one’s health.

### Statistical Analysis

Descriptive statistics were performed on participant characteristics collected at enrollment. Repeated-measures analyses of covariance calculated the adjusted mean changes and compared baseline and 6-month follow-up scores for the five QOL domains. Caregiver’s age, sex, race (reference group: white/Caucasian), ethnicity (reference group: Hispanic), levels of risk (RAM), and total number of therapeutic contacts were included as covariates. An effect size (*d* = [post-test mean − pretest mean]/pretest standard deviation) was computed for each outcome and categorized as small (*d* = 0.2), medium (*d* = 0.5), or large (*d* = 0.8; Cohen, 1988). After controlling for covariates (e.g., age, gender, race, ethnicity, risk level, sum of contacts), an ancillary analysis was conducted using generalized linear mixed modeling (GLMM) to assess changes in the QOL of caregivers who enrolled twice in the program. GLMMs allow inclusion of data from all participants, even if some participants were not seen at every assessment, and do not require equal intervals between assessments (Petranovich et al., 2015). GLMMs are also likelihood-based approaches that provide unbiased estimates of the intervention effects under the assumption of missing at random (Smith et al., 2015). All analyses were performed using SPSS version 25.0 (IBM Corp., Armonk, NY).

## Results

### Participant Characteristics

**Figure 1.**
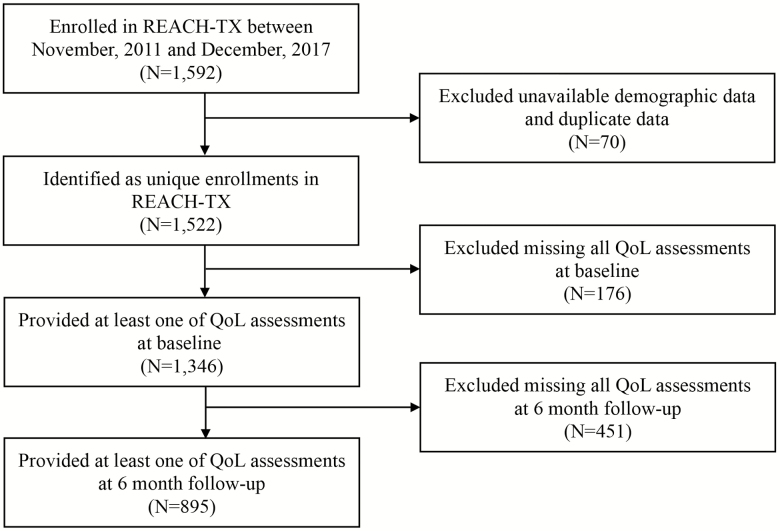
Flow chart.

As shown in Figure 1, a total of 1,592 caregivers enrolled in REACH-TX between November 2011 and December 2017. Among them, 1,522 unique caregivers provided demographic data and baseline assessments and enrolled in the program. Participants missing data from all five QOL domains at baseline (*n* = 176) and 6-month follow-up (*n* = 451) were excluded from the statistical analysis. Fifty-three caregivers who enrolled in the REACH-TX program twice were used in the ancillary analysis.

Table 2 shows characteristics of 1,522 unique caregivers at first enrollment. The average age was 62.9 years (*SD* = 13.4). The majority were female (78.1%), white/Caucasian (80.4%), and non-Hispanic/Latino (88.4%). More than 17% were black/African American (17.7%). Over 27% of caregivers (27.8%) were low risk, 70.4% were medium risk, and only 1.8% were high risk. The average therapeutic contacts for low, medium, and high risk were 6.50, 7.79, and 10.00, respectively.

**Table 2.** Characteristics of REACH-TX Caregivers at First Enrolment (*N* = 1,522)

Participant characteristics	
Age, mean (*SD*)	62.9 (13.4)
Gender, *n* (%)	
Female	1,188 (78.1)
Male	334 (21.9)
Race, *n* (%)	
White/Caucasian	1,224 (80.4)
Black/African American	269 (17.7)
Asian	15 (1.0)
Other^a^	10 (0.7)
Unknown	4 (0.4)
Ethnicity, *n* (%)	
Hispanic/Latino	176 (11.6)
Non-Hispanic/Latino	1,346 (88.4)
Risk Appraisal Measure (RAM), *n* (%)	
Low (RAM score: 0–11)	421 (27.8)
Medium (RAM score: 12–27)	1,066 (70.4)
High (RAM score: 28–40)	28 (1.8)
Number of therapeutic contacts, mean (*SD*)^b^	
Low	6.50 (3.48)
Medium	7.79 (3.12)
High	10.00 (3.61)

*Notes:* ^a^Other includes American Indian/Alaska Native, Native Hawaiian/Other Pacific Islander, and multirace. ^b^Caregivers who received a minimal number of contacts at each risk level were included.

### Changes in Five Domains of QOL From Baseline to 6-Month Follow-up

Table 3 shows changes in the five QOL domains from baseline to 6-month follow-up. Caregivers’ depression decreased by 2.38 (*p* < .001), which represents a small effect size (*d* = −0.42). Caregiver burden decreased by 4.36 (*p* < .001) with medium effect size (*d* = −0.52). Social support improved 1.42 (*p* < .001) with small effect size (*d* = 0.28). Significant improvement occurred in self-care (*p* < .01) and behavioral problems (*p* = .03). The effect sizes were small (*d* = 0.10 for self-care; *d* = 0.10 for behavioral problems), indicating caregivers were able to better take care of their own health and perceived substantial improvement in the loved one’s health after the completion of REACH-TX.

**Table 3.** Changes in Five Domains of Quality of Life From Baseline to 6-Month Follow-up

Domain	*N* ^a^	Baseline mean (± *SD*)	Follow-up mean (± *SD*)	Estimated mean difference	*SE*	95% confidence interval	*p*-value	Effect size
Depression	835	9.82 (6.36)	7.44 (6.10)	−2.38	0.21	(−2.80, −1.96)	<.001	−0.42
Caregiver burden	817	21.13 (9.13)	16.77 (9.06)	−4.36	0.33	(−5.00, −3.72)	<.001	−0.52
Social support	835	13.22 (5.40)	14.65 (5.38)	1.42	0.17	(1.09, 1.76)	<.001	0.28
Self-care	835	6.35 (1.98)	6.53 (1.87)	0.18	0.07	(0.06, 0.31)	<.01	0.10
Behavioral problem	741	0.58 (0.94)	0.67 (1.05)	0.10	0.05	(0.01, 0.19)	.03	0.10

*Note:* ^a^Number of cases varies due to missing cases.

**Figure 2.**
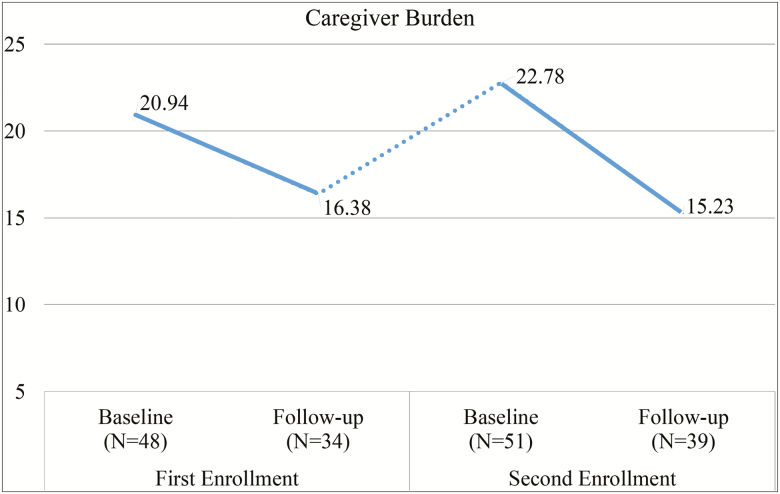
Changes in caregiving burden over time. Number of cases varies due to missing cases.

**Figure 3.**
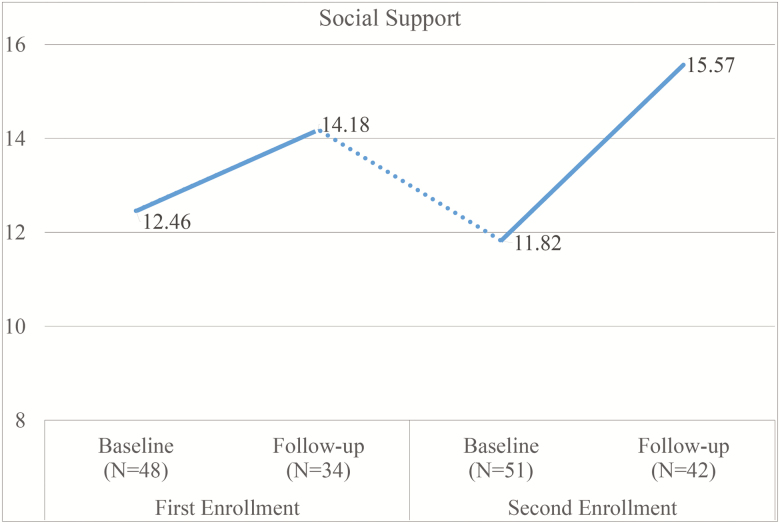
Changes in social support over time. Number of cases varies due to missing cases.

After controlling for covariates, an ancillary analysis (i.e., GLMM) of caregivers who enrolled a second time in REACH-TX revealed the adjusted mean levels of caregiver burden and social support changed significantly over the four assessments. Figures 2 and 3 show the changes of caregiver burden and social support over time. For participants who enrolled twice, the decline in caregiver burden from baseline of their second enrollment to follow-up of their second enrollment (mean difference = −7.55, *p* = .003) is significantly higher than the decline from baseline of their first enrollment to follow-up of their first enrollment (mean difference = −4.56, *p* = .276). The increase in social support from baseline of their second enrollment to follow-up of their second enrollment (mean difference = 3.75, *p* < .001) also is significantly higher than the increase from baseline of their first enrollment to follow-up of their first enrollment (mean difference = 1.72, *p* = .536). The remaining three domains did not show significant changes over time but showed similar patterns.

## Discussion

Family caregiving research has yielded numerous evidence-based interventions that support the use of a variety of therapeutic strategies to improve caregivers’ QOL. Far too few of these proven interventions have advanced into systematic translation and implementation studies, leaving unclear the impact of evidence-based interventions in real-world care and service settings. This study provides evidence of the impact of a community-based service program modeled on a leading evidence-based intervention for family caregivers for persons living with Alzheimer’s disease and dementia. Evaluation data of a modified version of the REACH II intervention (REACH-TX) presented here supports the feasible translation and implementation of REACH II by a community-based agency that yields positive benefits for family caregivers. Family caregivers for persons living with Alzheimer’s disease and dementia (i.e., care recipients) were positively affected by REACH-TX as demonstrated by change over time on the core QOL instruments used in the clinical trial of the evidence-based caregiver education and skill-training program. The findings of this study are consistent with other translational efforts (REACH OUT Program: Burgio et al., 2009; Community REACH: Czaja et al., 2018; REACH VA programs: Nichols, Martindale-Adams, Burns, Graney, & Zuber, 2011). Similar levels of effect sizes on the outcome assessments indicate that this translation effort of the community-based support service program is substantially beneficial to participants. Importantly, the community-based agency (AANCC) is in its seventh year of delivering the program, suggesting that REACH-TX is not only effective but also sustainable.

This study adds unique information to what is known about the process of translating caregiver interventions into community-based services in three domains previously designated as key elements in the translation process. The three domains of translational efforts informed by this REACH-TX study are streamlining dose and intensity, modifying treatment manuals, and evaluating translational activities (Gitlin et al., 2015). It appears that dose and intensity of the therapeutic service can be modified based on a risk-assessment approach. REACH-TX provides two to six in-home therapeutic sessions and at least two telephone contacts between sessions over a period of 6 months based on the caregiving situation as described by the caregiver. Basing services on need rather than a standard protocol as done in the original REACH II clinical trial helps to align REACH-TX with other service models commonly used by community-based organizations. REACH-TX modified the original treatment manuals into an adult-learner format that allowed for tailoring of educational materials to meet the needs of the family caregiver. The *A Caregiver’s Notebook* included educational materials, interactive modules, and worksheets corresponding to all target areas of the original REACH II. This approach also provided the family caregiver with similar information delivered by a computerized telephone system used in a clinical trial (Belle et al., 2006). Lastly and most importantly, REACH-TX conducted translational activities by utilizing and assessing outcomes from the original REACH II clinical trial. As shown in the results, REACH-TX demonstrated effectiveness in improving all five QOL domains. Moreover, ongoing communication between the community agency and an evaluation team played a significant role in improving the DCSs’ implementation skills as well as sustaining a partnership with the local funding agency for longer periods of time (i.e., 7 years).

Noteworthy from this study is the real-world approach of allowing caregivers to experience multiple exposures to the program. Not surprisingly, the 6-month treatment delivery approach was not sufficient for some caregivers. Multiple exposures to REACH-TX were found to be beneficial in reducing burden and improving social support in long-term caregiving experiences. This result demonstrates that the benefits on caregiver QOL associated with the program will diminish over time for some caregiver, and emphasizes the importance of sustaining a program over time as individuals may seek out helps from multiple exposures to the program. Aside from the fact that repeat enrollees are older than nonrepeat enrollees; however, it is unclear what other factors influenced caregivers’ decision to enroll in the program more than once. Those caregivers may have sought more support for a variety of reasons, and they may have been more engaged in other activities and services as caregiving tasks for care recipients required more commitment from caregivers. Future research may consider factors that affect caregiver interest, level of commitment to program activities, and needs; incorporate them into the program; and provide a different mechanism of support.

Although findings from this study have important implications for community-based support services for family caregivers, studies that occur within a community-based service-delivery model are associated with limitations. First, the dropout rate from enrollment to the 6-month follow-up was larger than expected in traditional research studies due to characteristics of the service-delivery setting that independently implemented the program. Unlike traditional and pragmatic clinical trials in which informed consent constitutes enrollment and baseline data collection immediately follows, the community-based agency-based enrollment on a telephone screening and then collected baseline assessment data on a home visit that also served as an initial therapeutic visit of the program. Caregivers who initially enrolled but later declined a home visit had no baseline data and were not provided the REACH-TX program. Other characteristics contributed to missing follow-up data. Of note, the data collection model was new to the existing culture of the community-based organizations. In the early years of implementation of REACH-TX, staff were more motivated to enroll and serve caregivers than to complete follow-up assessments once services have been delivered, as reported to the evaluation team from leaders of the agency. The effort to improve follow-up assessment collection was reflected in annual reports to the funding agency who worked with the agency. In other words, a change in evaluation criteria (i.e., 50% follow-up assessment required in performance standard evaluation) contributed to decreasing the missing rate in the follow-up measurements from 54.2% in 2012 to 36.7% in 2017. We, however, noticed significant differences in age and racial/ethnic groups between participants providing follow-up outcomes and participants missing all follow-up outcomes. The younger participants and African American participants tended to have more missing follow-up outcomes than their counterparts. Strategies to address this issue within these populations should be considered in future practice. Second, the service-delivery model of the organizations dictated that all interactions with the family caregiver be conducted by the staff of the agency, which limited the number of variables collected. Thus, the authors were not able to include specific caregiver characteristics, such as the caregiver’s health, number of caregiving hours and duration, or available resources (e.g., income level). Additional contextual information and caregiver characteristics may have provided insight into the compounding effects of community-based services on family caregivers’ QOL. Third, recruiting participants relied on referrals from local organizations through collaborations, which may cause self-selection bias. However, in the real-world setting, agencies are designed to serve those seeking services. Fourth, it was the staff of the community-based organization who collected the data for use in this study to track service delivery and for evaluation reports to the funder; therefore, the authors were not completely involved in quality assurance measures to ensure fidelity to standard interview practices. A standard assessment to monitor fidelity would be developed and utilized in future studies. Fifth, as dementia progresses over time, caregiving experiences may be challenging to improve, which would consequently affect a caregiver’s burden and social support. Nevertheless, it might be a concern that the participants who enrolled in the program twice might be exposed to repeated measures. Future studies should consider strategies to detect the possible effects of score inflation. Lastly, one limitation of the measurements used in this study is the low reliability of self-care. These items assess a caregiver’s diligence in looking after his or her own health. The internal consistency reported here is similar to the original REACH II trial (.49 for female; .57 for male). Developing a more reliable measure to assess a caregiver’s health should be a goal of future caregiving studies.

### Conclusion

Outcomes of this study suggest that REACH-TX is a feasible and sustainable evidence-based program to support family caregivers for people living with dementia (i.e., care recipients). Furthermore, this study contributes to providing valuable information on process of how care practices can be successfully delivered in a community setting and on the efforts and features of working outside the context of a highly structured RCT (Gitlin et al., 2015; Maslow, 2012). Partnerships between agencies that fund local services appear to be a formula for making REACH-TX available for family caregivers. Moreover, in partnership with a nonprofit health care organization, an evaluation model that incorporated community-based participatory research methods (Minkler & Wallerstein, 2008) provided valuable evaluation data back to the funding agency (United Way of Tarrant County) who, based on outcome data, continued to fund the program. Additional translational research of evidence-based interventions is needed in real-world settings to establish the science around comprehensive assessment of service delivery (i.e., what is provided and how much), participant characteristics (i.e., living arrangements, availability of other supports, overall resource needs of the family), and reasons for terminating services. These efforts would maximize limited resources to support the growing number and the increased diversity of family caregivers for those living with Alzheimer’s disease and dementia, and they would promote mutual benefits for researchers and communities.

## Funding

This work was supported by the United Way and Area Agency on Aging of Tarrant County and the National Institute on Aging of the National Institutes of Health under award number 1RC4AG038183-01.

## Supplementary Material

igz022_Suppl_Supplementary_Material
