# Erratum to: Evaluation of REACH-TX: A Community-Based Approach to the REACH II Intervention

**DOI:** 10.1093/geroni/igz041

**Published:** 2019-11-14

**Authors:** Jinmyoung Cho, Susanna Luk-Jones, Donald R Smith, Alan B Stevens

**Affiliations:** 1 Center for Applied Health Research, Baylor Scott & White Health, Temple, Texas; 2 Center for Population Health & Aging, Texas A&M University Health Science Center, College Station; 3 Department of Environmental and Occupational Health, Texas A&M University Health Science Center, College Station; 4 Family Care Services Unit, Alzheimer’s Association North Central Texas Chapter, Fort Worth; 5 Area Agency on Aging, United Way of Tarrant County, Fort Worth, Texas; 6 College of Medicine, Texas A&M University Health Science Center, College Station

The Translational Significance statement was incorrect as originally published. It has now been corrected to:

REACH II, a six-month intervention shown to reduce caregiver burden in randomized control clinical trials, was modified in a community setting (named REACH-TX) to include the reduced in-person and telephone contacts between the caregiver and the Dementia Care Specialist. The REACH-TX was found to be effective in improving the quality of life including reducing caregiver burden when administered by a community agency.

