# Supplementary material for: Evaluation of REACH-TX: A Community-Based Approach to the REACH II Intervention
Source: Innov Aging. 2019 Aug 23;3(3):igz022. doi: 10.1093/geroni/igz022 (PMC6735956; doi:10.1093/geroni/igz022)
Supplement: igz022_Suppl_Supplementary_Material [file igz022_suppl_supplementary_material.docx]

Supplementary Table 1

*REACH-TX Intervention Components and Activities*

| **INTERVENTION COMPONENT & TOPICS** | **ACTIVITIES** | | | |
| --- | --- | --- | --- | --- |
|  | **Assessment & Feedback** | Occurs across all intervention topics though the family profile and DCS observations | | |
|  | **Support** | Occurs across all intervention topics as DCS encouragement, validation, and empowerment | | |
|  | **Education** | **Skills-training** | **Problem-solving** | **Referral** |
| **Safety**   - CR Driving - CR Supervision Gaps - CR Wandering Risk - CR Fall Risk - Home Safety Hazards | - Basics of dementia and the impact of symptoms on safety - Common safety hazards and steps to take to mitigate them |  | - Assist the CG in overcoming obstacles to reducing safety hazards addressed in the Family Profile | - Information on Safe Return program for wandering |
| **Social Support**   - CG support making decisions, managing caregiving tasks, and handling difficult situations - Reduce CG social isolation - Assistance with both caregiving and non-caregiving tasks | - Importance of engaging social support | - Identify help needed as a CG - Identify help CG can offer as a friend | - Assist the CG in overcoming obstacles to identifying and accessing possible sources of social support | - Information on local caregiver support groups - Information on mental health counseling for significant emotional distress |
| **Managing Stress**   - Enhance CG stress management skills | - Education on stress and impact on health | - Specific stress management techniques including stretching and signal breath - Use of the stress diary to track the impact of stress management techniques on stress levels | - Assist the CG in using the stress diary to manage stress | - Information on respite and respite providers |
| **Pleasant Activities**   - Develop strategies to do more small, everyday pleasant activities both with and without the CR to improve stress management and emotional wellbeing - Use pleasant activities with the CR to improve the quality of the caregiving relationship | - Importance of incorporating enjoyable activities into daily life | - Pleasant event scheduling both with the care recipient and without | - Assist the caregiver in identifying pleasant activities, making arrangements to engage in pleasant activities, and overcoming barriers to engaging in pleasant activities |  |
| **Healthy Living**   - Organize health information for better care management and to aid interactions with health care - Engage in regular physical activity to promote health | - Importance of maintaining physical health | - Use of the America's SENIOR Health Guide to organize medical information - Warm-up for physical activity with stretching | - Assist the caregiver in overcoming obstacles to pursuing healthy behaviors | - Information on community resources for physical activity and chronic condition management |
| **Understanding Feelings**   - Stop and identify thoughts causing unpleasant feelings - Challenge and replace negative thoughts with something more positive - Attend to difference in feelings after replacing unhelpful thoughts with more helpful ones | - The role of thoughts on mood and emotional state | - Use of the Thought Record to identify and track thoughts contributing to negative emotions - Use of techniques to challenge negative thinking | - Assist the caregiver with any difficulties using the Thought Record tool |  |
| **Skillful Communication**   - Improve CG communication with the CR - Develop CG skills to help CR communicate | - Impact of dementia on CR receptive and expressive communication | - Communication strategies to make CG more easily understood by the CR - Communication strategies for CG to help CR communicate his/her needs | - Assist the caregiver in overcoming specific communication challenges |  |
| **Problem Behaviors**   - Improve CG daily care skills to avoid problem behaviors - Develop CG skills to identify and avoid triggers of CR behaviors - Develop CG skills to respond effectively to problem behaviors to mitigate their impact | - Relationship between memory loss and problem behavior - Best practices for assisting with daily care activities | - Use of ABC’s of behavior management to identify and track behaviors (antecedents) and the impact of various responses (consequences) | - Assist CG with finding creative solutions to avoid behavior triggers - Assist CG in finding alternative responses to problem behaviors |  |
| **Legal And Medical Information**   - Acquire legal, medical, and financial paperwork | - Various legal, medical, and financial documents and their uses |  | - Assist CG in finding resources recommended in the Family Profile | - Information on community benefits that may assist with legal, medical, and financial planning and provision |
